# Clinical and genetic differences between bipolar disorder type 1 and 2 in multiplex families

**DOI:** 10.1038/s41398-020-01146-0

**Published:** 2021-01-11

**Authors:** Jose Guzman-Parra, Fabian Streit, Andreas J. Forstner, Jana Strohmaier, Maria José González, Susana Gil Flores, Francisco J. Cabaleiro Fabeiro, Francisco del Río Noriega, Fermin Perez Perez, Jesus Haro González, Guillermo Orozco Diaz, Yolanda de Diego-Otero, Berta Moreno-Kustner, Georg Auburger, Franziska Degenhardt, Stefanie Heilmann-Heimbach, Stefan Herms, Per Hoffmann, Josef Frank, Jerome C. Foo, Lea Sirignano, Stephanie H. Witt, Sven Cichon, Fabio Rivas, Fermín Mayoral, Markus M. Nöthen, Till F. M. Andlauer, Marcella Rietschel

**Affiliations:** 1 Department of Mental Health, University Regional Hospital of Málaga, Institute of Biomedicine of Málaga (IBIMA), Málaga, Spain; 2 Department of Genetic Epidemiology in Psychiatry, Central Institute of Mental Health, Medical Faculty Mannheim, Heidelberg University, Mannheim, Germany; 3 Centre for Human Genetics, University of Marburg, Marburg, Germany; 4 Institute of Human Genetics, University of Bonn, School of Medicine & University Hospital Bonn, Bonn, Germany; 5 Department of Mental Health, Hospital of Puerto Real, Cádiz, Spain; 6 Department of Mental Health, University Hospital of Reina Sofia, Cordoba, Spain; 7 Department of Mental Health, Hospital of Jaen, Jaen, Spain; 8 Department of Mental Health, Hospital of Jerez de la Frontera, Jerez de la Frontera, Spain; 9 Department of Mental Health, Hospital Punta de Europa, Algeciras, Spain; 10 Unidad de Gestión Clínica del Dispositivo de Cuidados Críticos y Urgencias del Distrito Sanitario Málaga-Coin-Guadalhorce, Málaga, Spain; 11 Department of Personality, Assessment and Psychological Treatment, University of Malaga, Institute of Biomedicine of Málaga (IBIMA), Málaga, Spain; 12 Department of Neurology, Goethe University Medical School, Frankfurt am Main, Germany; 13 Department of Biomedicine, University of Basel, Basel, Switzerland; 14 Institute of Neuroscience and Medicine (INM-1), Research Center Jülich, Jülich, Germany; 15 Department of Neurology, Klinikum rechts der Isar, School of Medicine, Technical University of Munich, Munich, Germany

**Keywords:** Bipolar disorder, Clinical genetics

## Abstract

The two major subtypes of bipolar disorder (BD), BD-I and BD-II, are distinguished based on the presence of manic or hypomanic episodes. Historically, BD-II was perceived as a less severe form of BD-I. Recent research has challenged this concept of a severity continuum. Studies in large samples of unrelated patients have described clinical and genetic differences between the subtypes. Besides an increased schizophrenia polygenic risk load in BD-I, these studies also observed an increased depression risk load in BD-II patients. The present study assessed whether such clinical and genetic differences are also found in BD patients from multiplex families, which exhibit reduced genetic and environmental heterogeneity. Comparing 252 BD-I and 75 BD-II patients from the Andalusian Bipolar Family (ABiF) study, the clinical course, symptoms during depressive and manic episodes, and psychiatric comorbidities were analyzed. Furthermore, polygenic risk scores (PRS) for BD, schizophrenia, and depression were assessed. BD-I patients not only suffered from more severe symptoms during manic episodes but also more frequently showed incapacity during depressive episodes. A higher BD PRS was significantly associated with suicidal ideation. Moreover, BD-I cases exhibited lower depression PRS. In line with a severity continuum from BD-II to BD-I, our results link BD-I to a more pronounced clinical presentation in both mania and depression and indicate that the polygenic risk load of BD predisposes to more severe disorder characteristics. Nevertheless, our results suggest that the genetic risk burden for depression also shapes disorder presentation and increases the likelihood of BD-II subtype development.

## Introduction

Bipolar disorder (BD) is a severe, highly heritable mental disorder characterized by fluctuations in mood state and energy with recurring episodes of depression altering with episodes of mania or hypomania. Its early onset, chronicity, high prevalence (of approximately 1%), and lack of optimal treatment renders it one of the world’s most disabling conditions^1^. BD is etiologically heterogeneous, and the current diagnostic classification describes two major subtypes, BD type I (BD-I) and II (BD-II), which differ by the absence of full-blown manic episodes in BD-II. BD-I can be diagnosed based on a single manic episode, yet, in most cases, depressive episodes also occur. BD-II is diagnosed if at least one hypomanic and one depressive episode have occurred. Hypomanic episodes are, by definition, clinically less severe than manic ones, potentially have a shorter duration, are not characterized by a marked impairment in social or occupational functioning, and do not require hospitalization; the occurrence of any psychotic symptoms qualifies an episode as manic^2^.

Until recently, it was thought that both BD subtypes could be classified along a spectrum of the affective disorders defined by the extent and severity of mood elevation (i.e., major depressive disorder (MDD) < BD-II < BD-I). This concept is in line with findings of BD-I patients suffering from more psychotic^3–6^ and melancholic symptoms^3^, more hospitalizations^6–8^, and more severe and widespread impairment of cognitive functions^9^. It is also in line with the observation in several previous studies, that risk for either subtype of BD is higher for those with relatives affected by BD-I (compared to MDD and BD-II)^2,10,11^. However, this concept has been challenged by a series of studies which report a higher total number of episodes^12–14^, increased comorbidity with anxiety disorders^8,13,14^ and personality disorders^8,15^, as well as lower functioning^15^ and quality of life^16^ in BD-II compared to BD-I patients, although these findings were not consistent across studies^7,8,17,18^.

Recent large-scale formal and molecular genetic studies reported a higher heritability of BD-I than of BD-II^11,19–21^ and a high genetic correlation between BD-I and BD-II^19–21^. While the difference in heritability and the high genetic correlation between the subtypes may argue in favor of a severity continuum^21^, other findings instead point to partially distinct genetic etiologies of BD-I and BD-II. In contrast to other previous studies^2,10,11^, a large-scale Swedish registry-based family study found that relatives of BD patients showed the highest risk for the respective BD subtype: relatives of patients with BD-I had an increased risk for BD-I compared to BD-II and relatives of patients with BD-II had a trend for a higher risk for BD-II^20^. Different patterns of familial co-aggregation, genetic correlation, and polygenic risk score (PRS) analyses of both subtypes with other psychiatric disorders, in particular of BD-I with schizophrenia (SCZ) and of BD-II with MDD, underline potential molecular differences between the two subtypes^11,19–24^.

Clinical differences between BD-I and BD-II cases have rarely been studied in multiplex families with a high density of BD cases. An advantage of such multiplex families is the reduced genetic and environmental heterogeneity compared to unrelated case/control cohorts^25^. In a previous study on BD multiplex families, Frankland et al.^26^ described more mixed states in BD-II and more psychomotor retardation as well as more psychotic features of depressive episodes in BD-I cases. We have previously shown that, compared to unaffected family members and unrelated controls, BD patients from multiplex families have a high genetic risk load specifically for BD^27,28^. Furthermore, family members also had a higher genetic risk load for SCZ and, to a lesser degree, for MDD than unrelated controls^28^. Therefore, we hypothesize that a correlation between the genetic risk burden for psychiatric disorders and disease severity exists in these families.

The present study had three aims: first, to examine whether BD-I and BD-II patients from multiplex families with a high density of BD differ regarding their clinical course, the symptoms presenting during episodes, and psychiatric comorbidities. Second, to analyze whether the genetic risk burden for BD, SCZ, and MDD differed between the subtypes. Third, to investigate whether the PRS for BD, SCZ, and MDD were higher in patients showing more severe symptoms.

## Materials and methods

### Sample description

The study subjects are part of the Andalusian Bipolar Family (ABiF) study, which gathered data from 100 Spanish families with at least two cases of BD per family and has been described elsewhere^29^. In the present analyses, 327 BD patients from 98 families were included (BD-I *n* = 252, BD-II *n* = 75). The individual families were unrelated to each other, and the average pedigree contained 11.8 family members (SD = 7.6), including 3.3 (SD = 2.5) BD and 2.0 (SD = 2.2) MDD patients (Supplementary Table S1). Each family contained at least two BD patients. In addition, two pedigrees contained one schizophrenia patient each. The sample included 58.4% females and had an average age of 48.21 years (SD = 17.22; range=18–96; age refers to the age either at the interview or at the reported time of the patient’s death). The predominant level of education was primary school or less (70.6%) and was lower in patients with an earlier decade of birth. Diagnosis and clinical data were based on the Schedule for Affective Disorders and Schizophrenia (SADS)^30^, the Structured Clinical Interview for DSM IV Axis I Disorders (SCID-I)^31^, the Family Informant Schedule and Criteria (FISC)^32^, and on clinical records. The protocol of the structured diagnostic interview was modified to assess symptoms during lifetime episodes and not only those present in the most severe episode. Diagnoses were given by two trained clinicians using the best estimate approach. For 46 BD-I and 2 BD-II patients, no interview was available, and diagnoses were based on data assessed through best informants only. The local ethics committees of five Andalusian provinces approved the study (Comités de ética de la investigación provincial de Cádiz, Córdoba, Granada, Jaén, and Málaga), and all participants gave their written informed consent.

### Genotyping and calculation of PRSs

Genetic information was available for 156 individuals (BD-I *n* = 115, BD-II *n* = 41) from 33 families. Genome-wide genotyping was carried out using the Illumina Infinium PsychArray BeadChip (PsychChip). All quality control (QC) and imputation procedures have been described previously^28^. In brief, QC and population substructure analyses were performed in PLINK v1.9^33^, as described in the Supplementary Methods. The data were imputed to the 1000 Genomes phase 3 reference panel using SHAPEIT and IMPUTE2^34–36^. The imputed dataset contained 8,628,089 variants.

For the calculation of PRSs^37^, SNP weights were estimated using the PRS-CS method^38^ with default parameters (see the Supplementary Methods). This method employs Bayesian regression to infer PRS weights while modeling the local linkage disequilibrium patterns of all SNPs using the EUR super-population of the 1000 Genomes reference panel, without requiring the calculation of several PRS using different *p*-value thresholds. The global shrinkage parameter φ was determined automatically (PRS-CS-auto; BD: φ = 1.22 × 10^−4^, MDD: φ = 1.26 × 10^−4^, BD: φ = 1.47 × 10^−4^). The PRSs were calculated, using these weights, in PLINK v1.90b6.13 on imputed dosage data^33^. As training data, we used summary statistics of genome-wide association studies (GWAS) by the Psychiatric Genomics Consortium (PGC) containing 20,352 cases and 31,358 controls for BD^19^, 170,756 cases and 329,443 controls for MDD^39^, and 33,640 cases and 43,456 controls for SCZ^40^. As the ABiF cohort was part of the PGC BD GWAS^19^, Spanish samples were excluded from the BD training GWAS to avoid bias caused by sample overlap.

### Generalized estimating equations

We used generalized estimating equations (GEEs), calculated using the package *geepack*^41^ in *R* v4.0.2, to analyze whether BD-I and BD-II differed regarding their demographic information, clinical course, symptoms presenting during episodes, and psychiatric comorbidities. GEEs are suitable for correlated data in samples with a family structure. An exchangeable correlation structure was selected, as is appropriate for the family structure of the sample. To allow for a stable fit, models were only calculated for variables that contained ≥10 individuals within each category (e.g., symptoms present and not present). For variables that did not meet this requirement, no test coefficients are provided in Tables 1–4. Secondary analyses including only probands with a personal interview are provided in Supplementary Table S2.

**Table 1:** Sociodemographic characteristics of BD-I (*n* = 252) and BD-II (*n* = 75) patients.

Variable	BD-I	*N*	BD-II	*N*	*P*	OR	95% CI
Age	48 (14)	245	40 (13)	75	0.024	1.02	1.00–1.04
Gender (female)	144 (57.14)	252	47 (62.67)	75	0.49	0.80	0.43–1.50
Marital status (separated, divorced, or single) *Reference:* married or widowed	73 (29.08)	251	17 (22.67)	75	0.31	1.40	0.73–2.69
Educational level (secondary school or university degree) *Reference:* Primary or <4 years of school	66 (26.19)	252	29 (39.19)	74	0.38	0.74	0.38–1.45

*BD* bipolar disorder, *n* valid sample size for the variable, *OR* odds ratio, *95% CI* 95% confidence interval. Bonferroni-corrected threshold for significance: α = 0.05/37 = 1.35 × 10^−03^.

Age refers to the age either at the interview or at the reported time of the patient’s death. Age and gender were used as independent variables, with BD type as the dependent variable. Marital status and educational levels were used as dependent variables, with BD type as the independent variable. We used the following covariates: marital status, sex; educational level, sex and decade of birth. The age is described by median and median absolute deviation, sample size (*N*) and percentage are provided for categorical variables.

**Table 2:** Differences in the clinical course between BD-I and BD-II patients.

Variable	BD-I	*N*	BD-II	*N*	*P*	β	SE
Age at first episode (years)	21 (5)	233	21 (6)	75	0.52	0.10	0.15
Age at first manic episode (years)	25 (7)	230	26 (8)	70	0.75	0.05	0.14
Age at first depressive episode (years)	22 (5.5)	230	21 (6)	75	0.59	0.08	0.16
Duration of illness (years)	23 (11)	233	16 (9)	75	2.83 × 10^−03^	0.36	0.12
Duration of depressive episodes (weeks)	16 (8)	247	12 (8)	75	0.14	0.21	0.14
Number of depressive episodes/illness duration	0.86 (0.57)	229	0.78 (0.58)	75	0.27	0.17	0.16
Number of (hypo)manic episodes/illness duration	0.52 (0.38)	232	0.77 (0.65)	75	0.029	−0.35	0.16
Number of suicide attempts/illness duration (*median*)	0 (0)	233	0 (0)	75	0.046	0.20	0.10
Number of suicide attempts/illness duration (*mean*)	0.04 (0.14)	233	0.02 (0.08)	75	0.046	0.20	0.10

Variable	BD-I	*N*	BD-II	*N*	*P*	OR	95% CI
Depressive polarity of the first episode	102 (43.78)	233	38 (50.7)	75	0.22	0.75	0.48–1.18
ECT during depressive episodes	9 (3.64)	247	1 (1.33)	75	0.42	2.41	0.28–20.95
Medication during depressive episodes	237 (95.18)	249	61 (81.33)	75	0.010	3.93	1.38–11.20
Hospitalization during depressive episodes	50 (20.16)	248	3 (4.00)	75	2.45 × 10^−03^	6.41	1.93–21.35
**Incapacity during depressive episodes**	158 (69.30)	228	33 (45.83)	72	**7.07** **×** **10**^**−04**^	**2.51**	**1.47–4.28**
Suicide attempted (ever)	65 (25.79)	252	11 (14.67)	75	0.040	2.46	1.04–5.79
Serious or extreme suicide attempt	18 (7.14)	252	2 (2.66)	75	0.13	3.44	0.70–17.03
Seasonality	139 (58.65)	237	36 (49.31)	73	0.043	1.73	1.02–2.95

*BD* bipolar disorder, *SE* standard error, *N* sample size for the variable (before correction for covariates), *OR* odds ratio, *95% CI* 95% confidence interval, *ECT* electroconvulsive therapy. Bonferroni-corrected threshold for significance: α = 0.05/37 = 1.35 × 10^−03^.

Quantitative variables were analyzed in linear, dichotomous variables in logistic models. BD type was the independent variable in all models. We used the following covariates: quantitative variables, sex; dichotomous variables, sex and age. All quantitative variables have been transformed for the analyses using inverse rank-based normalization to generate normally distributed residuals. The numbers of episodes and suicide attempts were divided by the illness duration before the transformation. See Supplementary Table S6 for analyses of these variables not divided by illness duration. All quantitative variables are described by median and median absolute deviation of untransformed variables, sample size (*N*) and percentage are provided for categorical variables. For the number of suicide attempts, mean and standard deviation are also provided. Significant variables are labeled in bold font.

**Table 3:** Comparison of the symptoms profiles during lifetime between BD-I and BD-II patients.

Symptoms during manic/hypomanic episodes	BD-I	*N*	BD-II	*N*	*P*	OR	95% CI
Hyperactivity	252 (100)	252	75 (100)	75			
Talkativeness	252 (100)	252	73 (97.33)	75			
Flight of idea	236 (99.58)	237	68 (97.14)	70			
Inflated grandiosity	248 (99.20)	250	73 (97.33)	75			
Decreased need for sleep	251 (99.60)	252	72 (96.00)	75			
**Inattention**	239 (95.60)	250	64 (85.33)	75	**7.19** **×** **10**^**−04**^	**4.79**	**1.93–11.88**
**Reckless behavior**	166 (66.13)	251	9 (12.16)	73	**3.95** **×** **10**^**−13**^	**13.48**	**6.68–27.20**
Delusions	217 (87.85)	247	0	75			
Hallucinations	20 (8.16)	245	0	75			

Symptoms during depressive episodes	BD-I	*N*	BD-II	*N*	*P*	OR	95% CI
Appetite change	240 (96.38)	249	70 (93.33)	75	0.25	1.80	0.66–4.94
Loss of appetite or weight	209 (90.87)	230	67 (93.05)	72	0.61	0.76	0.27–2.15
Increased appetite or weight	15 (6.52)	230	5 (6.94)	72	0.91	1.05	0.46–2.37
Sleep change	245 (98.39)	249	73 (97.33)	75			
Early morning awakening	176 (79.64)	221	48 (67.60)	71	0.030	1.82	1.06–3.11
Hypersomnia	5 (2.16)	231	2 (2.78)	72			
Psychomotor alterations	243 (97.59)	249	72 (96.00)	75			
Fatigue or tiredness	248 (99.60)	249	74 (98.67)	75			
Diminished libido	236 (97.52)	242	71 (94.96)	74			
Guilt	215 (86.34)	249	63 (84.00)	75	0.97	1.02	0.36–2.91
Difficult thinking or indecisiveness	241 (96.79)	249	72 (96.00)	75	0.15	2.18	0.75–6.34
Suicidal ideation	222 (89.15)	249	57 (72.00)	75	4.09 × 10^−03^	2.51	1.34–4.70
Loss of pleasure	221 (95.26)	232	69 (95.83)	72	0.98	1.02	0.24–4.36
Lack of reactivity	149 (64.78)	230	44 (61.11)	72	0.81	1.07	0.63–1.80
Different feeling of sadness	206 (94.49)	218	68 (94.44)	72	0.88	1.10	0.32–3.72
Morning worsening	175 (76.75)	228	46 (63.89)	72	0.025	1.86	1.08–3.21
Excessive guilt	196 (84.48)	232	59 (81.94)	72	0.65	1.24	0.49–3.13
Leaden paralysis	103 (44.59)	231	24 (33.33)	72	0.05	1.81	1.00–3.27
Delusions	35 (14.11)	248	3 (4.00)	75	3.56 × 10^−03^	4.80	1.67–13.78
Hallucinations	28 (11.29)	248	2 (2.67)	75	0.021	5.57	1.29–24.04

*BD* bipolar disorder, *n* valid sample size for the variable, *OR* odds ratio, *95% CI* 95% confidence interval. Bonferroni-corrected threshold for significance: α = 0.05/37 = 1.35 × 10^−03^.

BD type was the independent variable in all models. Sample size (*N*) and percentage are provided for categorical variables. Significant variables are labeled in bold font. To allow for a stable fit, models were only calculated for variables that contained ≥10 individuals per variable category. Variables without test coefficients (P, OR, CI) did not meet this requirement.

**Table 4:** Comparison of comorbidities between BD-I and BD-II patients.

Comorbidity	BD-I	*N*	BD-II	*N*	*P*	OR	95% CI
Alcohol abuse	30 (11.90)	252	5 (6.67)	75	0.32	1.72	0.59–5.00
Drug abuse	15 (5.95)	252	6 (8.00)	75	0.87	1.09	0.38–3.13
Alcohol dependence	7 (2.78)	252	3 (4.00)	75	0.46	0.65	0.20–2.07
Drug dependence	3 (1.19)	252	0	75			
Panic disorder	6 (2.38)	252	0	75			
Obsessive compulsive disorder	2 (0.80)	251	0	75			
Phobia	2 (0.80)	251	0	75			
Somatoform disorder	2 (0.83)	242	0	71			
Cyclothymic personality	26 (10.32)	252	7 (9.33)	75	0.97	1.02	0.43–2.41
Any comorbid disorder	71 (28.17)	252	17 (22.67)	75	0.31	1.38	0.74–2.57

*BD* bipolar disorder, *N* valid sample size for the variable, *OR* odds ratio, *95% CI* 95% confidence interval. Bonferroni-corrected threshold for significance: α = 0.05/37 = 1.35 × 10^−03^.

BD type was the independent variable in all models. Sample size (*N*) and percentage are provided for categorical variables. To allow for a stable fit, models were only calculated for variables that contained ≥10 individuals per variable category. Variables without test coefficients (P, OR, CI) did not meet this requirement.

Sex and age were used as covariates in all analyses of non-sociodemographic, dichotomous variables. Only sex was used for quantitative clinical variables that already contained a temporal component. Quantitative variables were transformed using inverse rank-based normal transformation. The number of episodes and suicide attempts were divided by the illness duration before the transformation. Here, the illness duration is defined as the difference between the age and the age at BD onset. This ratio allowed for a better normalization than was observed when transforming the variables by themselves and adding the illness duration as a covariate to the model. Secondary analyses were conducted with illness duration as a covariate instead of dividing the numbers by the duration (Supplementary Table S3).

### Correction for multiple testing

To establish an appropriate correction threshold for multiple comparisons using Bonferroni’s method, the number of independent tests reported in Tables 1–4 was estimated using principal component analysis (PCA) in *R* using the function *prcomp*. Of the 42 variables analyzed, the first 37 components jointly explained >99% (99.3%) of the phenotypic variance (Supplementary Fig. S1). We thus set the significance threshold to α = 0.05/37 = 1.35 × 10^−03^. We used a more liberal threshold to select variables for genetic analyses: the first nine components explained >50% (53.1%) of the phenotypic variance, corresponding to a significance threshold of α = 0.05/9 = 5.56 × 10^−03^.

### Analyses of PRSs

PRS were analyzed to compare the polygenic risk burden for BD, MDD, and SCZ between BD types and to explore the influence of PRS on the clinical characteristics. We analyzed the association of PRS with all dichotomous phenotypes showing a *p* < 5.56 × 10^−03^ in GEE analyses (see above). PRS analyses were carried out as previously described^28^ in *R* using the function *glmm.wald* of the package *GMMAT*^42^, fitted by maximum likelihood using Nelder-Mead optimization. Family structure was modeled as a random effect, with a genetic relationship matrix calculated on pruned genotype data using GEMMA^43^.

PRSs were transformed by Z-score standardization before the analyses. Age and sex were used as fixed-effects covariates in all analyses. We used Bonferroni’s method to correct for three tests (α = 0.05/3 = 0.0167) in the analyses of PRS with BD type and for 18 tests (three PRS and six variables) in the analyses of PRS with clinical course and symptoms variables (α = 0.05/18 = 2.78 × 10^−03^). Based on a previous report^19^, we expected higher SCZ PRS in BD-I cases and higher MDD PRS in BD-II cases. Here, also one-sided *p*-values are reported.

### Permutation analyses

All association *p*-values were validated using permutation analyses. Here, the null distribution of test statistics was empirically determined by repeating regression analyses with a random sampling of phenotype data. To calculate a *p*-value, the number of tests were counted where a model with a random association showed the same or a more extreme, i.e., smaller *p*-value than the correct, non-randomized model; this number was divided by the total number of tests. Results from these permutation analyses are, together with the respective number of permutations, presented in Supplementary Tables S3-S6. Some permutation *p*-values were higher than the *p*-values directly estimated with GEE models, but all variables that were significant in the primary GEE analyses showed a permutation *p* ≤ 2.66 × 10^−03^ (Supplementary Table S4). Typically, the permutation *p*-values of PRS analyses were lower than the *p*-values from GMMAT Wald tests (Supplementary Tables S5-S6).

### Power analysis

We conducted power analyses for classical, *p*-value threshold-based PRS using AVENGEME^44^. Because PRS calculated by PRS-CS have a higher power^45^, we assume that these estimates constitute lower boundaries of our real statistical power. According to these analyses, we had a power of 0.64, 0.73, and 0.39 for analyses of BD-I *vs*. BD-II with PRS for BD, SCZ, and MDD, respectively (Supplementary Table S7). For the quantitative traits, the power was 0.91, 0.95, and 0.65 for BD, SCZ, and MDD, respectively.

## Results

The sociodemographic variables did not differ significantly between family members diagnosed with BD-I and BD-II (Table 1). Among the variables related to the clinical course of disorder (Table 2), BD-I patients more frequently showed incapacity during depressive episodes (odds ratio (OR) = 2.51, 95% confidence interval (CI) = 1.47-4.28, *p* = 7.07 × 10^−04^) after Bonferroni correction for 37 independent tests (α = 1.35 × 10^−03^; 37 principal components explain 99.3% of the phenotypic variance). Furthermore, BD-I patients showed a longer average illness duration (β = 0.36 standard deviations, SE = 0.12, *p* = 2.83 × 10^−03^) and more hospitalizations during depressive episodes (OR = 6.41, CI = 1.93-21.35, *p* = 2.45 × 10^−03^), yet those two analyses did not withstand correction for multiple testing.

During (hypo)manic episodes, BD-I patients exhibited significantly more inattention (OR = 4.79, CI = 1.93–11.88, *p* = 7.19 × 10^−04^; Table 3) and reckless behavior (OR = 13.48, CI = 6.68–27.20, *p* = 3.95 × 10^−13^). The *p*-value of reckless behavior was higher in the permutation analysis but remained significant (*p* = 1.26 × 10^−04^, Supplementary Table S4). During depressive episodes, suicidal ideation (OR = 2.51, CI = 1.34–4.70, *p* = 4.09 × 10^−03^) and delusions (OR = 4.80, CI = 1.67–13.78, *p* = 3.56 × 10^−03^) were observed more frequently in BD-I patients, but these associations were not significant after correction for multiple testing. BD-I and BD-II patients did not differ significantly regarding the examined comorbidities with other mental disorders (Table 4).

When only analyzing patients with a personal interview, incapacity during depressive episodes and reckless behavior remained significant (Supplementary Table S2). In these secondary analyses, inattention did not remain significant after correction for multiple testing. By contrast, medication and hospitalization during depressive episodes, both only nominally significant in the primary analysis, were significant after correction for multiple testing in the secondary analysis.

**Fig. 1:**
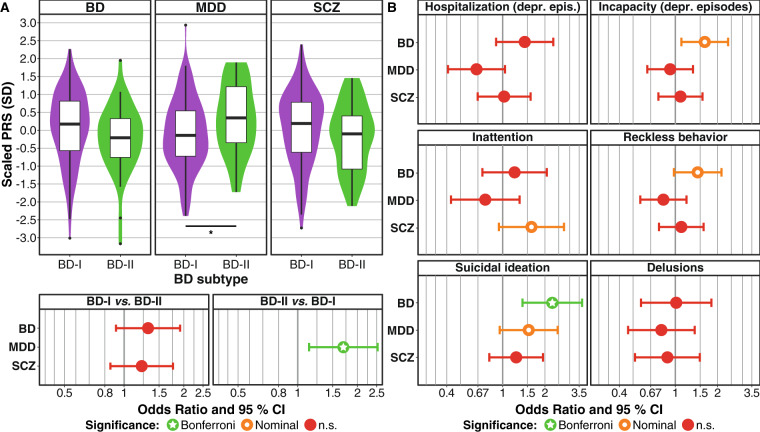
Polygenic risk score analyses in a subset of 156 family members. **A** BD-II patients showed a significantly higher MDD PRS than BD-I cases. SD standard deviation. Results from a logistic mixed regression model using PRS as predictors, BD type as the outcome, and sex and age as covariates (for full results see Supplementary Table S5). Comparisons significant after Bonferroni correction for three tests (α = 0.05/3 = 0.0167) are marked with an asterisk. For the SCZ PRS, the one-sided hypothesis was that BD-I cases have higher PRS; for the MDD PRS, the one-sided hypothesis was that BD-II cases show higher PRS. **B** Analysis of dichotomous clinical course and symptom variables with *p* < 5.56 × 10^−03^ in the phenotypic analyses (see Tables 2–3). Results from a logistic mixed regression model using PRS as predictors, symptoms as the outcome, and sex and age as covariates (for full results see Supplementary Table S6). Bonferroni-corrected threshold for significance: α = 0.05/(6 × 3) = 2.78 × 10^−03^. For all PRS, the one-sided hypothesis was that the symptom severity increases with the PRS. PRS polygenic risk score, BD bipolar disorder, MDD major depressive disorder, SCZ schizophrenia, 95% CI 95% confidence interval, depr. epis*.* depressive episodes.

Next, we analyzed PRS in 156 of the 327 patients for which genetic data were available. In this smaller subset, the statistical power was reduced compared to the phenotype-level analyses (Supplementary Table S7). In our analysis whether PRS for BD^19^, MDD^39^, or SCZ^40^ differed between BD subtypes (Fig. 1A, Supplementary Table S4), BD-II cases showed a significantly higher MDD risk burden after correction for three tests (OR = 1.70, CI = 1.14–2.53, *p* = 9.11 × 10^−03^, *p*_one-sided_ = 4.55 × 10^−03^). No significant differences were observed for BD and SCZ PRS.

We finally analyzed whether PRS were higher in patients showing a more severe clinical course or more severe symptoms. We conducted these analyses for the six dichotomous variables differing between BD subtypes in primary analyses, prioritized using a *p*-value threshold of α = 5.56 × 10^−03^ (nine components explain 53.1% of the phenotypic variance). BD PRS were significantly higher in patients showing suicidal ideation after Bonferroni correction for 18 tests (OR = 2.25, CI = 1.38-3.67, *p* = 1.11 × 10^−03^, *p*_1-sided_ = 5.53 × 10^−04^, Fig. 1B, Supplementary Table S5). Furthermore, BD PRS were higher in patients with incapacity during depressive episodes, yet this association did not remain significant after correction for multiple testing.

## Discussion

The present study investigated phenotypic and genetic differences between BD-I and BD-II patients of BD multiplex families: First, patients diagnosed with BD-I showed both more severe manic episodes, with frequent inattention and reckless behavior, and more serious depressive episodes, which were often characterized by incapacity. Second, BD-II patients exhibited a significantly higher genetic MDD risk load than family members diagnosed with BD-I. Third, the genetic risk burden for BD was significantly associated with suicidal ideation.

It is not surprising that we found reckless behavior to be more frequent in BD-I than in BD-II cases, as it constitutes one of the main reasons for hospitalization. Previous studies have already described reckless behavior and inattention as features of BD-I^14,46^.

An increased incapacity of BD-I patients during depressive episodes was previously reported in a study conducted in unrelated BD patients^7^. Besides increased occupational impairment, further severity-related measures were described in this previous study, e.g., an increased need for medication and psychiatric hospitalizations, both of which only achieved nominal significance in the present study but were significant in our secondary analyses of patients with a personal interview. Furthermore, a tendency of BD-I patients to display more psychotic symptoms during their depressive episodes did not remain significant after correction for multiple testing in the present study. Previous findings regarding differences in the number of depressive episodes between BD-I and BD-II patients—not significant in the present study—were mixed: Studies in multiplex families found either no difference^47^ or more depressive episodes for BD-I^26^. Conversely, studies in unrelated patients in a clinical setting found more depressive episodes in BD-II patients^12,13^.

Berskon’s selection bias might have contributed to the observation of an increased severity and a higher number of depressive episodes in unrelated BD-II patients recruited from a clinical setting: It seems plausible that clinical samples of sporadic BD-II patients are enriched for patients suffering from more and more severe depressive episodes, for which they sought clinical help. Findings from the World Mental Health Survey Initiative (cross-sectional face-to-face interviews in 61,392 community-based adults from eleven countries)^1^ support this idea, finding that the severity of manic and depressive symptoms and of suicidal behavior increased monotonically from subthreshold BD to BD-II to BD-I.

Suicidal ideation was nominally associated with BD-I and can be considered a highly relevant indicator of mental disorder severity. Prior findings on the difference of suicidal ideations between BD-I and BD-II in samples of unrelated patients were inconsistent^3,7,14^. A large published epidemiological study investigating differences in suicidal ideation between the two subtypes in more than 1400 BD patients, found more suicidal ideation during depressive episodes in BD-I cases^7^. However, this difference was not observed in a previous study of multiplex families^26^. Published results are also mixed regarding lifetime suicide attempts^8,14,48–52^. A meta-analysis indicated possibly more suicide attempts in BD-I cases^53^, but this result was not significant (*p* = 0.07). Although suicide attempts occurred more frequently in BD-I than BD-II patients in the ABiF sample, this difference did not reach significance in the present study.

We have described previously that both affected and unaffected ABiF family members exhibit increased genetic risk loads for BD, MDD, and SCZ, compared to unaffected controls^28^. Within the families, BD patients showed higher BD PRS than unaffected family members^28^. In the present study, BD-II patients had a higher MDD risk load than BD-I patients, consistent with previous results from unrelated cases^19^. We observed a tendency for higher BD and SCZ PRS in BD-I patients, but both differences were not significant, possibly due to the small sample size, which impeded conclusive interpretations.

These findings do not unequivocally support the hypothesis that the BD genetic risk load shapes the development of either BD-II or BD-I along a severity continuum, i.e., that BDI cases carry more BD risk variants than BD-II cases. Instead, our previous^28^ and present results could suggest that, in the ABiF families, a general load of psychiatric, and especially BD, risk variants drive the overall BD vulnerability, while an increased MDD risk load may have shaped the family members’ BD presentation towards the BD-II subtype. The association of the BD PRS with suicidal ideation indicates that the polygenic makeup might, beyond its contribution to categorical subtypes of BD, shape the patients’ individual disorder manifestation on a symptom level. However, more studies with larger samples are needed to confirm these hypotheses.

There were several limitations of this study. We analyzed lifetime symptoms and not only symptoms during the worst episode, which should be kept in mind when interpreting and comparing the results to other studies. Furthermore, the small sample size, especially in the genetic analyses, limits the generalizability of results obtained and increases the likelihood of type II errors. Thus, these results need to be interpreted with caution. However, the family-based design and the homogeneity of the sample may have improved statistical power. Still, future studies in the ABiF study and other family cohorts should aim to analyze larger samples. Moreover, the present study only analyzed common genetic variants, and rare variants could also have contributed to clinical differences of BD patients in the ABiF multiplex families^54,55^. Accordingly, a notable disadvantage of studying multiplex families is that they may harbor more rare variants than patients recruited from the general population. In addition, their common variant patterns and shared environmental factors may differ from those observed in unrelated case/control samples. We analyzed a multi-generational study, and demographic factors, especially the educational system and the access to it, have changed during the 20^th^ century in Spain. In earlier generations, the average educational level was lower, and more family members were married. The GWAS used for calculating the BD PRS contained over four times as many BD-I than BD-II cases^19^ and, therefore, the BD PRS was likely more sensitive for the genetic architecture of BD-I than for BD-II.

In summary, the present study compared clinical, symptomatological, and genetic features of BD-I and BD-II patients from BD multiplex pedigrees. The finding that BD-I patients showed more severe symptoms during their manic as well as during their depressive episodes, and the association of the BD PRS with suicidal ideation, are in line with a genetic severity continuum in multiplex BD families. The observation of a lower genetic risk load for MDD in BD-I patients, however, points to a more complex situation: It indicates that the individual polygenic risk load for different psychiatric disorders may influence the development of either BD-I or BD-II and the associated symptoms. Future studies should aim to replicate these results and examine the underlying mechanisms, e.g., biological pathways or potentially protective effects of high MDD PRS on the development of pronounced manic symptoms.

## Supplementary information

Supplementary Material
