# Supplementary material for: Clinical and genetic differences between bipolar disorder type 1 and 2 in multiplex families
Source: Transl Psychiatry. 2021 Jan 11;11:31. doi: 10.1038/s41398-020-01146-0 (PMC7801527; doi:10.1038/s41398-020-01146-0)
Supplement: Supplementary file 1 — Supplementary Material [file 41398_2020_1146_MOESM1_ESM.docx]

**Clinical and genetic differences between bipolar disorder type 1 and 2 in multiplex families – Supplementary Material**

**Supplementary Methods**

# Genetic quality control (QC)

QC of genotype data (Infinium PsychArray BeadChip (PsychChip)) was conducted in PLINK v1.90b3.36.

**Sequence of QC steps:**

Before QC: 395 individuals and 588,454 variants

1. Removal of SNPs with call rates <98% or a MAF <1%
2. Check for individuals with genotyping rates <98% (*none removed*)
3. Check for sex mismatches (*none removed*)
4. Removal of non-autosomal variants
5. Removal of SNPs with call rates <98%, a MAF <1%, or Hardy-Weinberg Equilibrium (HWE) test *p*-values <1×10^-6^
6. Removal of A/T and G/C SNPs
7. Update of variant IDs and positions to the IDs and positions in the 1000 Genomes Phase 3 reference panel
8. Alignment of alleles to the reference panel
9. Removal of duplicated variants and of variants not present in the reference panel

After QC: 395 individuals and 258,046 variants

Of these, 156 individuals were retained for the present study (after the imputation).

# Imputation of genotype data

Genotypes were aligned to the 1000 Genomes Phase 3 reference panel using SHAPEIT v2 (r837) and PLINK v1.90b3.36. Pre-phasing (haplotype estimation) was conducted for each chromosome separately using SHAPEIT with the *--duohmm* option.

Imputation was performed using IMPUTE2 v2.3.2 in 5 Mbp chunks with 500 kbp buffers, filtering out variants that are monomorphic in the EUR samples. Chunks with <51 genotyped variants or concordance rates <92 % were fused with neighboring chunks and re-imputed. Imputed variants with a MAF <1% or an INFO metric <0.8 were removed. Imputed variants after post-imputation QC: 8,628,089.

# PRS-CS parameters

We used the default PRS-CS parameters, as described in the manual (accessed on December 29^th^, 2019): <https://github.com/getian107/PRScs>

REFERENCE = ldblk_1kg_eur

PARAM_A = 1

PARAM_B = 0.5

PARAM_PHI = auto

MCMC_ITERATIONS = 1000

MCMC_BURNIN = 500

MCMC_THINNING_FACTOR = 5

BETA_STD = FALSE

**Supplementary Tables**

**Table S1:** Number of individuals, BD and MDD diagnosis per pedigree.

| Number of pedigrees per Andalusian province | N | N BD cases | N BD-I cases | N BD-II cases | N recurrent MDD cases | N single episode MDD cases | N healthy family members |
| --- | --- | --- | --- | --- | --- | --- | --- |
| 29 families from Málaga | 54 | 16 | 12 | 3 | 9 | 3 | 24 |
|  | 37 | 13 | 11 | 2 | 8 | 1 | 15 |
|  | 34 | 12 | 10 | 1 | 2 | 1 | 14 |
|  | 24 | 3 | 0 | 3 | 3 | 1 | 17 |
|  | 22 | 13 | 7 | 6 | 4 | 0 | 3 |
|  | 17 | 6 | 4 | 2 | 3 | 1 | 7 |
|  | 16 | 6 | 5 | 1 | 2 | 0 | 7 |
|  | 15 | 3 | 2 | 0 | 5 | 0 | 6 |
|  | 15 | 6 | 5 | 0 | 3 | 0 | 6 |
|  | 14 | 4 | 3 | 1 | 1 | 0 | 9 |
|  | 14 | 4 | 2 | 1 | 0 | 1 | 8 |
|  | 13 | 4 | 2 | 1 | 1 | 0 | 6 |
|  | 13 | 5 | 2 | 2 | 3 | 0 | 4 |
|  | 13 | 2 | 2 | 0 | 1 | 0 | 9 |
|  | 12 | 3 | 1 | 2 | 3 | 4 | 2 |
|  | 11 | 2 | 2 | 0 | 2 | 0 | 7 |
|  | 10 | 5 | 5 | 0 | 1 | 0 | 3 |
|  | 10 | 5 | 4 | 1 | 0 | 0 | 5 |
|  | 10 | 3 | 1 | 2 | 0 | 0 | 5 |
|  | 10 | 2 | 1 | 1 | 3 | 0 | 5 |
|  | 9 | 2 | 1 | 0 | 3 | 0 | 3 |
|  | 8 | 4 | 1 | 3 | 1 | 0 | 2 |
|  | 8 | 3 | 3 | 0 | 1 | 0 | 0 |
|  | 8 | 3 | 3 | 0 | 1 | 1 | 3 |
|  | 8 | 2 | 2 | 0 | 0 | 0 | 6 |
|  | 6 | 3 | 3 | 0 | 0 | 0 | 3 |
|  | 5 | 4 | 3 | 0 | 1 | 0 | 1 |
|  | 5 | 3 | 2 | 0 | 0 | 0 | 1 |
|  | 4 | 2 | 2 | 0 | 0 | 0 | 2 |
| 14 families from Granada | 22 | 4 | 2 | 2 | 8 | 1 | 9 |
|  | 21 | 5 | 1 | 3 | 4 | 0 | 9 |
|  | 20 | 7 | 5 | 2 | 0 | 3 | 10 |
|  | 18 | 3 | 1 | 2 | 4 | 1 | 7 |
|  | 15 | 4 | 2 | 1 | 2 | 1 | 8 |
|  | 14 | 3 | 3 | 0 | 3 | 0 | 8 |
|  | 14 | 2 | 1 | 0 | 4 | 1 | 5 |
|  | 10 | 3 | 1 | 2 | 1 | 0 | 6 |
|  | 10 | 2 | 2 | 0 | 5 | 1 | 2 |
|  | 10 | 2 | 1 | 0 | 3 | 0 | 3 |
|  | 9 | 2 | 2 | 0 | 2 | 0 | 5 |
|  | 8 | 4 | 3 | 0 | 0 | 0 | 3 |
|  | 7 | 2 | 2 | 0 | 3 | 0 | 2 |
|  | 7 | 2 | 2 | 0 | 0 | 0 | 5 |
| 27 families from Córdoba | 23 | 5 | 3 | 2 | 1 | 1 | 16 |
|  | 17 | 6 | 4 | 2 | 2 | 1 | 7 |
|  | 17 | 5 | 4 | 1 | 4 | 0 | 8 |
|  | 16 | 3 | 2 | 1 | 4 | 0 | 9 |
|  | 15 | 4 | 4 | 0 | 3 | 1 | 7 |
|  | 15 | 6 | 3 | 2 | 1 | 1 | 7 |
|  | 14 | 3 | 1 | 2 | 2 | 0 | 8 |
|  | 14 | 2 | 1 | 0 | 4 | 0 | 8 |
|  | 11 | 4 | 4 | 0 | 0 | 1 | 6 |
|  | 11 | 3 | 3 | 0 | 1 | 1 | 6 |
|  | 10 | 4 | 2 | 2 | 2 | 0 | 4 |
|  | 10 | 2 | 2 | 0 | 2 | 0 | 4 |
|  | 9 | 2 | 1 | 1 | 2 | 0 | 4 |
|  | 9 | 2 | 2 | 0 | 1 | 0 | 2 |
|  | 9 | 2 | 1 | 0 | 2 | 0 | 4 |
|  | 8 | 3 | 2 | 1 | 1 | 1 | 2 |
|  | 8 | 2 | 2 | 0 | 0 | 0 | 6 |
|  | 8 | 2 | 1 | 1 | 1 | 0 | 3 |
|  | 8 | 2 | 1 | 1 | 0 | 0 | 6 |
|  | 7 | 2 | 2 | 0 | 3 | 0 | 2 |
|  | 7 | 2 | 1 | 0 | 2 | 0 | 3 |
|  | 6 | 2 | 2 | 0 | 0 | 0 | 4 |
|  | 6 | 2 | 2 | 0 | 1 | 1 | 2 |
|  | 6 | 2 | 1 | 0 | 1 | 1 | 2 |
|  | 5 | 2 | 2 | 0 | 2 | 0 | 3 |
|  | 3 | 2 | 1 | 0 | 1 | 0 | 0 |
|  | 3 | 2 | 1 | 0 | 0 | 0 | 1 |
| 19 families from Jaén | 30 | 8 | 5 | 3 | 2 | 1 | 17 |
|  | 18 | 5 | 5 | 0 | 4 | 0 | 9 |
|  | 15 | 2 | 2 | 0 | 5 | 0 | 7 |
|  | 13 | 4 | 2 | 2 | 0 | 0 | 9 |
|  | 12 | 4 | 3 | 1 | 0 | 0 | 7 |
|  | 11 | 5 | 5 | 0 | 0 | 0 | 6 |
|  | 11 | 3 | 1 | 2 | 2 | 0 | 4 |
|  | 11 | 3 | 3 | 0 | 2 | 0 | 6 |
|  | 11 | 3 | 1 | 2 | 0 | 0 | 8 |
|  | 11 | 2 | 1 | 1 | 2 | 0 | 7 |
|  | 11 | 2 | 2 | 0 | 0 | 1 | 8 |
|  | 11 | 2 | 2 | 0 | 0 | 0 | 9 |
|  | 10 | 3 | 3 | 0 | 1 | 0 | 5 |
|  | 10 | 3 | 3 | 0 | 1 | 0 | 5 |
|  | 10 | 2 | 2 | 0 | 1 | 0 | 7 |
|  | 9 | 4 | 4 | 0 | 0 | 0 | 5 |
|  | 9 | 2 | 1 | 0 | 1 | 0 | 6 |
|  | 8 | 2 | 0 | 1 | 0 | 0 | 6 |
|  | 7 | 2 | 1 | 0 | 1 | 0 | 3 |
| 9 families from Cádiz | 12 | 6 | 5 | 1 | 1 | 0 | 5 |
|  | 10 | 3 | 3 | 0 | 1 | 0 | 5 |
|  | 8 | 4 | 2 | 2 | 1 | 0 | 3 |
|  | 3 | 3 | 3 | 0 | 0 | 0 | 0 |
|  | 3 | 2 | 2 | 0 | 0 | 0 | 1 |
|  | 3 | 2 | 2 | 0 | 0 | 0 | 1 |
|  | 2 | 2 | 2 | 0 | 0 | 0 | 0 |
|  | 2 | 2 | 2 | 0 | 0 | 0 | 0 |
|  | 2 | 2 | 1 | 0 | 0 | 0 | 0 |

**Table S2:** Results from phenotypic GEE analyses of patients with a personal interview.

These analyses correspond to the results presented in Tables 1-4, please see the legends of these tables for further details. Here, patients without a personal interview were excluded. To allow for a stable fit, models were only calculated for variables that contained ≥10 individuals within each category. Less variables than in the primary analyses met this criterion. All *p*-values are two-sided. Bonferroni-corrected threshold for significance: α=0.05/37=1.35×10^-03^, significant variables are labeled in bold font. Analysis: Demogr., sociodemographic characteristics (Table 1); Clinical, clinical course (Table 2); Man. ep., symptoms during manic/hypomanic episodes (Table 3); Depr. ep., symptoms during depressive episodes (Table 3); Comorb., comorbidities (Table 4).

| Analysis | Variable | BD-I | N | BD-II | N | P | OR | 95% CI |
| --- | --- | --- | --- | --- | --- | --- | --- | --- |
| Demogr. | Age | 45 (13) | 205 | 40 (13) | 73 | 0.23 | 1.01 | 0.99-1.03 |
| Demogr. | Gender (female) | 120 (58.55) | 206 | 46 (63.01) | 73 | 0.58 | 0.84 | 0.45-1.57 |
| Demogr. | Marital status | 69 (33.50) | 206 | 17 (23.29) | 73 | 0.12 | 1.64 | 0.88-3.07 |
| Demogr. | Educational level | 62 (30.10) | 206 | 28 (38.89) | 72 | 0.47 | 0.78 | 0.39-1.55 |
| **Analysis** | **Variable** | **BD-I** | **N** | **BD-II** | **N** | **P** | **β** | **SE** |
| Clinical | Age at first episode (years) | 21 (5) | 204 | 21 (6) | 73 | 0.80 | 0.04 | 0.16 |
| Clinical | Age at first manic episode (years) | 24 (6) | 202 | 26 (8) | 69 | 0.85 | 0.03 | 0.15 |
| Clinical | Age at first depressive episode (years) | 21 (5) | 201 | 21 (6) | 73 | 0.84 | 0.03 | 0.16 |
| Clinical | Duration of illness (years) | 21 (10) | 204 | 16 (8) | 73 | 0.028 | 0.28 | 0.13 |
| Clinical | Duration of depressive episodes (weeks) | 20 (12) | 202 | 12 (8) | 73 | 0.094 | 0.24 | 0.14 |
| Clinical | Number of depressive episodes  / illness duration | 0.86 (0.57) | 201 | 0.74 (0.54) | 73 | 0.28 | 0.17 | 0.15 |
| Clinical | Number of (hypo)manic episodes / illness duration | 0.49 (0.35) | 204 | 0.71 (0.61) | 73 | 0.018 | -0.37 | 0.16 |
| Clinical | Number of suicide attempts / illness duration | 0 (0) | 205 | 0 (0) | 73 | 0.052 | 0.20 | 0.10 |
| **Analysis** | **Variable** | **BD-I** | **N** | **BD-II** | **N** | **P** | **OR** | **95% CI** |
| Clinical | Depressive polarity of the first episode | 97 (47.55) | 204 | 38 (52.05) | 73 | 0.35 | 0.80 | 0.51- 1.27 |
| Clinical | ECT during depressive episodes | 7 (3.48) | 201 | 1 (1.37) | 73 | NA | NA | NA |
| **Clinical** | **Medication during depressive episodes** | **195 (96.05)** | **203** | **59 (80.82)** | **73** | **1.22×10^-03^** | **5.31** | **1.93-14.62** |
| **Clinical** | **Hospitalization during depressive episodes** | **49 (24.26)** | **202** | **3 (4.11)** | **73** | **7.65×10^-04^** | **7.39** | **2.31-23.69** |
| **Clinical** | **Incapacity during depressive episodes** | **123 (67.21)** | **183** | **32 (45.71)** | **70** | **5.13×10^-04^** | **2.57** | **1.51- 4.38** |
| Clinical | Suicide attempted (ever) | 56 (27.18) | 206 | 11 (15.07) | 73 | 0.049 | 2.38 | 1.00- 5.66 |
| Clinical | Serious or extreme suicide attempt | 14 (6.80) | 206 | 2 (2.74) | 73 | 0.11 | 4.18 | 0.72-24.47 |
| Clinical | Seasonality | 135 (66.50) | 203 | 36 (50.0) | 72 | 1.72×10^-03^ | 2.30 | 1.37- 3.87 |
| Man. ep. | Inattention | 195 (94.66) | 206 | 62 (84.93) | **73** | 1.91×10^-03^ | 4.35 | 1.72-10.99 |
| **Man. ep.** | **Reckless behavior** | **131 (63.59)** | **206** | **8 (10.96)** | **73** | **1.35×10^-12^** | **15.01** | **7.10-31.74** |
| Depr. ep. | Appetite change | 194 (95.57) | 203 | 68 (93.15) | 73 | 0.30 | 1.67 | 0.64- 4.39 |
| Depr. ep. | Loss of appetite or weight | 179 (90.40) | 198 | 66 (92.96) | 71 | 0.64 | 0.77 | 0.26- 2.29 |
| Depr. ep. | Increased appetite or weight | 15 (7.61) | 197 | 5 (7.04) | 71 | 0.81 | 1.11 | 0.48- 2.54 |
| Depr. ep. | Early morning awakening | 155 (78.28) | 198 | 48 (67.61) | 71 | 0.045 | 1.74 | 1.01- 2.98 |
| Depr. ep. | Guilt | 173 (85.22) | 203 | 61 (83.56) | 73 | 0.85 | 1.11 | 0.39- 3.16 |
| Depr. ep. | Difficult thinking / indecisiveness | 195 (96.06) | 203 | 70 (95.89) | 73 | 0.29 | 1.89 | 0.58- 6.11 |
| **Analysis** | **Variable** | **BD-I** | **N** | **BD-II** | **N** | **P** | **OR** | **95% CI** |
| Depr. ep. | Suicidal ideation | 178 (87.68) | 203 | 55 (75.34) | 73 | 7.11×10^-03^ | 2.40 | 1.27- 4.55 |
| Depr. ep. | Loss of pleasure | 188 (94.95) | 198 | 68 (95.77) | 71 | 0.92 | 0.92 | 0.19- 4.38 |
| Depr. ep. | Lack of reactivity | 121 (61.11) | 198 | 43 (60.56) | 71 | 0.96 | 1.01 | 0.58- 1.76 |
| Depr. ep. | Different feeling of sadness | 187 (94.44) | 198 | 67 (94.37) | 71 | 0.93 | 1.06 | 0.29- 3.88 |
| Depr. ep. | Morning worsening | 147 (74.24) | 198 | 45 (63.38) | 71 | 0.051 | 1.76 | 1.00- 3.11 |
| Depr. ep. | Excessive guilt | 164 (82.83) | 198 | 58 (81.69) | 71 | 0.78 | 1.15 | 0.43- 3.02 |
| Depr. ep. | Leaden paralysis | 95 (47.98) | 198 | 24 (33.80) | 71 | 0.035 | 1.87 | 1.05- 3.33 |
| Depr. ep. | Delusions | 33 (16.33) | 202 | 3 (4.11) | 73 | 2.32×10^-03^ | 4.83 | 1.75-13.31 |
| Depr. ep. | Hallucinations | 27 (13.37) | 202 | 2 (2.74) | 73 | 0.014 | 5.74 | 1.42-23.12 |
| Comorb. | Alcohol Abuse | 26 (12.62) | 206 | 5 (6.85) | 73 | 0.31 | 1.72 | 0.60- 4.91 |
| Comorb. | Drug Abuse | 15 (7.28) | 206 | 6 (8.22) | 73 | 0.80 | 1.15 | 0.39- 3.37 |
| Comorb. | Alcohol Dependence | 5 (2.43) | 206 | 3 (4.11) | 73 | NA | NA | NA |
| Comorb. | Cyclothymic personality | 25(12.14) | 206 | 7 (9.59) | 73 | 0.90 | 1.06 | 0.43- 2.59 |
| Comorb. | Any comorbid disorder | 65 (31.55) | 206 | 17 (23.29) | 73 | 0.28 | 1.40 | 0.76- 2.58 |

**Table S3:** Results from GEE analyses of quantitative variables with illness duration as a covariate.
Quantitative variables were analyzed in linear models with BD type as the independent variable and sex and illness duration as covariates. All variables have been transformed using inverse rank-based normalization. Here, the variables were **not** divided by the illness duration, instead the illness duration was used as a covariate. The variables are described by median and median absolute deviation of untransformed variables. For the number of suicide attempts, mean and standard deviation are also provided. SE=standard error, N=Sample size for the variable (before correction for covariates), P_permutation_ = *p*‑value after 100,000 permutations. Bonferroni-corrected threshold for significance: α=0.05/37=1.35×10^-03^. All *p*-values are two-sided.

| Variable | BD-I | N | BD-II | N | P | P_permutation_ | β | SE |
| --- | --- | --- | --- | --- | --- | --- | --- | --- |
| Number of depressive episodes | 20 (17) | 247 | 8 (7) | 75 | 0.14 | 0.16 | 0.19 | 0.13 |
| Number of (hypo)manic episodes | 10 (8) | 250 | 10 (9) | 75 | 0.085 | 0.102 | -0.22 | 0.13 |
| Number of suicide attempts (*median*) | 0 (0) | 252 | 0 (0) | 75 | 0.021 | 0.032 | 0.23 | 0.10 |
| Number of suicide attempts (*mean*) | 0.50 (1.14) | 252 | 0.28 (0.86) | 75 | 0.021 | 0.032 | 0.23 | 0.10 |
| Number of depressive episodes *(only personal interviews)* | 15 (12) | 203 | 8 (8) | 73 | 0.19 | 0.21 | 0.17 | 0.13 |
| Number of (hypo)manic episodes *(only personal interviews)* | 7 (5) | 206 | 10 (9) | 73 | 0.044 | 0.057 | -0.26 | 0.13 |
| Number of suicide attempts *(only personal interviews, median)* | 0 (0) | 206 | 0 (0) | 73 | 0.029 | 0.044 | 0.22 | 0.10 |
| Number of suicide attempts *(only personal interviews, mean)* | 0.55 (1.22) | 206 | 0.29 (0.87) | 73 | 0.029 | 0.044 | 0.22 | 0.10 |

**Table S4:** Results from permutation analyses of phenotypic variables.

These analyses correspond to Tables 2-4. All *p*-values are two-sided. Bonferroni-corrected threshold for significance: α=0.05/37=1.35×10^-03^ (bold font).

| Analysis | Variable | P | P_permutation_ | N_permutations_ |
| --- | --- | --- | --- | --- |
| Clinical course | Age at first episode (years) | 0.52 | 0.53 | 100,000 |
| Clinical course | Age at first manic episode (years) | 0.75 | 0.76 | 100,000 |
| Clinical course | Age at first depressive episode (years) | 0.59 | 0.60 | 100,000 |
| Clinical course | Duration of illness (years) | 2.83×10^-03^ | 6.71×10^-03^ | 1,000,000 |
| Clinical course | Duration of depressive episodes (weeks) | 0.13 | 0.15 | 100,000 |
| Clinical course | Number of depressive episodes  / illness duration | 0.14 | 0.16 | 100,000 |
| Clinical course | Number of (hypo)manic episodes  / illness duration | 0.27 | 0.29 | 100,000 |
| Clinical course | Number of suicide attempts / illness duration | 0.029 | 0.041 | 100,000 |
| Clinical course | Depressive polarity of the first episode | 0.22 | 0.24 | 100,000 |
| Clinical course | ECT during depressive episodes | 0.42 | 0.45 | 100,000 |
| Clinical course | Medication during depressive episodes | 0.010 | 0.013 | 100,000 |
| Clinical course | Hospitalization during depressive episodes | 2.45×10^-03^ | 5.85×10^-03^ | 1,000,000 |
| **Clinical course** | **Incapacity during depressive episodes** | **7.07×10^-04^** | 2.66×10^-03^ | 1,000,000 |
| Clinical course | Suicide attempted (ever) | 0.040 | 0.050 | 100,000 |
| Clinical course | Serious or extreme suicide attempt | 0.13 | 0.13 | 100,000 |
| Clinical course | Seasonality | 0.043 | 0.053 | 100,000 |
| **(Hypo)manic episodes** | **Inattention** | **7.19×10^-04^** | 4.60×10^-03^ | 1,000,000 |
| **(Hypo)manic episodes** | **Reckless behavior** | **3.95×10^-13^** | **1.26×10^-04^** | **1,000,000** |
| Depressive episodes | Appetite change | 0.25 | 0.25 | 100,000 |
| Depressive episodes | Loss of appetite or weight | 0.61 | 0.63 | 100,000 |
| Depressive episodes | Increased appetite or weight | 0.91 | 0.90 | 100,000 |
| Depressive episodes | Early morning awakening | 0.030 | 0.038 | 100,000 |
| Depressive episodes | Guilt | 0.97 | 0.97 | 100,000 |
| Depressive episodes | Difficult thinking or indecisiveness | 0.15 | 0.13 | 100,000 |
| Depressive episodes | Suicidal ideation | 4.09×10^-03^ | 7.73×10^-03^ | 1,000,000 |
| Depressive episodes | Loss of pleasure | 0.98 | 0.98 | 100,000 |
| Depressive episodes | Lack of reactivity | 0.81 | 0.82 | 100,000 |
| Depressive episodes | Different feeling of sadness | 0.88 | 0.83 | 100,000 |
| Depressive episodes | Morning worsening | 0.025 | 0.032 | 100,000 |
| Depressive episodes | Excessive guilt | 0.65 | 0.67 | 100,000 |
| Depressive episodes | Leaden paralysis | 0.049 | 0.059 | 100,000 |
| Depressive episodes | Delusions | 3.56×10^-03^ | 6.91×10^-03^ | 1,000,000 |
| Depressive episodes | Hallucinations | 0.021 | 0.022 | 100,000 |
| Comorbidity | Alcohol Abuse | 0.32 | 0.34 | 100,000 |
| Comorbidity | Drug Abuse | 0.87 | 0.87 | 100,000 |
| Comorbidity | Alcohol Dependence | 0.46 | 0.47 | 100,000 |
| Comorbidity | Cyclothymic personality | 0.97 | 0.97 | 100,000 |
| Comorbidity | Any comorbid disorder | 0.31 | 0.32 | 100,000 |

**Table S5:** Associations of polygenic risk scores (PRS) with BD subtype.

Results from a logistic mixed regression model using PRS as predictors, BD type as the outcome, and sex and age as covariates. N=115 BD-I and N=41 BD-II cases were used for this analysis. OR=odds ratio, CI=95% confidence interval. Bonferroni-corrected threshold for significance: α=0.05/3=0.01667, significant variables are labeled in bold font. For the SCZ PRS, the one-sided hypothesis was that BD-I cases have higher PRS; for the MDD PRS, the one-sided hypothesis was that BD-II cases show higher PRS. All permutation *p*-values (P_permutation_) are two-sided.

| Analysis | PRS | OR | 95% CI | P | P_1-sided_ | P_permutation_ | N_permutations_ |
| --- | --- | --- | --- | --- | --- | --- | --- |
| BD-I *vs.* BD-II | BD | 1.32 | 0.91-1.91 | 0.143 |  | 0.142 | 10,000 |
| BD-I *vs.* BD-II | SCZ | 1.23 | 0.85-1.76 | 0.270 | 0.135 | 0.267 | 10,000 |
| **BD-II *vs.* BD-I** | **MDD** | **1.70** | **1.14-2.53** | **9.11×10^-03^** | **4.55×10^-03^** | **7.04×10^-03^** | **100,000** |

**Table S6:** Associations of polygenic risk scores (PRS) with selected variables.

Analysis of dichotomous clinical course and symptom variables with *p*<5.56×10^-03^ in the phenotypic analyses. Results from a logistic mixed regression model using PRS as predictors, symptoms as the outcome, and sex and age as covariates. N=115 BD-I and N=41 BD-II cases were used for this analysis. OR=odds ratio, CI=95% confidence interval. Bonferroni-corrected threshold for significance: α=0.05/(6×3)=2.78×10^-03^, significant variables are labeled in bold font. For all PRS, the one-sided hypothesis was that the symptom severity increases with PRS. All permutation *p*-values (P_permutation_) are two-sided.

| Analysis | PRS | OR | 95% CI | P | P_1-sided_ | P_permutation_ | N_permutations_ |
| --- | --- | --- | --- | --- | --- | --- | --- |
| Hospitalization during depressive episodes | BD | 1.43 | 0.90-2.29 | 0.13 | 0.066 | 0.12 | **10,000** |
| Hospitalization during depressive episodes | MDD | 0.65 | 0.41-1.04 | 0.072 | 0.964 | 0.064 | **10,000** |
| Hospitalization during depressive episodes | SCZ | 1.02 | 0.66-1.58 | 0.92 | 0.46 | 0.91 | **10,000** |
| Incapacity during depressive episodes | BD | 1.62 | 1.10-2.37 | 0.014 | 6.80×10^-03^ | 0.010 | **100,000** |
| Incapacity during depressive episodes | MDD | 0.92 | 0.63-1.33 | 0.65 | 0.67 | 0.65 | **10,000** |
| Incapacity during depressive episodes | SCZ | 1.09 | 0.76-1.56 | 0.65 | 0.33 | 0.65 | **10,000** |
| Inattention | BD | 1.22 | 0.72-2.06 | 0.47 | 0.23 | 0.46 | **10,000** |
| Inattention | MDD | 0.75 | 0.43-1.32 | 0.32 | 0.84 | 0.31 | **10,000** |
| Inattention | SCZ | 1.60 | 0.94-2.73 | 0.083 | 0.042 | 0.074 | **10,000** |
| Reckless behavior | BD | 1.44 | 0.98-2.13 | 0.066 | 0.033 | 0.060 | **10,000** |
| Reckless behavior | MDD | 0.82 | 0.56-1.20 | 0.31 | 0.85 | 0.31 | **10,000** |
| Reckless behavior | SCZ | 1.10 | 0.77-1.59 | 0.60 | 0.30 | 0.60 | **10,000** |
| **Suicidal ideation** | **BD** | **2.25** | **1.38-3.67** | **1.11×10^-03^** | **5.53×10^-04^** | **6.00×10^-04^** | **100,000** |
| Suicidal ideation | MDD | 1.53 | 0.95-2.46 | 0.079 | 0.040 | 0.077 | **10,000** |
| Suicidal ideation | SCZ | 1.25 | 0.80-1.94 | 0.32 | 0.16 | 0.32 | **10,000** |
| Delusions | BD | 1.01 | 0.57-1.80 | 0.96 | 0.48 | 0.97 | **10,000** |
| Delusions | MDD | 0.80 | 0.46-1.37 | 0.41 | 0.80 | 0.40 | **10,000** |
| Delusions | SCZ | 0.88 | 0.52-1.49 | 0.63 | 0.69 | 0.63 | **10,000** |

**Table S7:** Results from the PRS power analysis using AVENGEME.

We used the function polygenescore() from AVENGEME

(<https://github.com/DudbridgeLab/avengeme/>) to estimate the statistical power of PRS analyses. For the logistic regression with BD-I *vs.* BD-II as the outcome, we assumed a prevalence of 2:3 of BD-I compared to BD-II. Since PRS calculated using PRS-CS have higher statistical power than classical PRS

(<https://www.medrxiv.org/content/10.1101/2020.09.10.20192310v1>), we assume these power estimates to constitute the lower boundary of our real power.

| Analysis | Training GWAS | Cases | Controls | SNP-based heritability | P-value threshold | N SNPs | Prevalence | Power |
| --- | --- | --- | --- | --- | --- | --- | --- | --- |
| BD-I *vs.* BD-II | BD | 20352 | 31358 | 0.23 | 0.01 | 13096 | 0.01 | 0.64 |
| BD-I *vs.* BD-II | SCZ | 33640 | 43456 | 0.24 | 0.01 | 17918 | 0.01 | 0.73 |
| BD-I *vs.* BD-II | MDD | 170756 | 329443 | 0.089 | 0.01 | 13935 | 0.02 | 0.39 |
| Quantitative | BD | 20352 | 31358 | 0.23 | 0.01 | 13096 | 0.01 | 0.91 |
| Quantitative | SCZ | 33640 | 43456 | 0.24 | 0.01 | 17918 | 0.01 | 0.95 |
| Quantitative | MDD | 170756 | 329443 | 0.089 | 0.01 | 13935 | 0.02 | 0.65 |

**Supplementary Figures**

**Supplementary Fig. S1:** The variance explained in the principal component analysis.

The plot shows the cumulative variance explained by the principal components calculated by principal component analysis of all scaled and centered variables (*R* function *prcomp*). The dark orange lines highlight the threshold used for Bonferroni correction in the phenotypic analyses (37 components jointly explain >99% of the variance). The light orange lines highlight the threshold used for selecting variables for genetic analyses (nine components jointly explain >50% of the variance).
